# Fast and Accurate Protein False Discovery Rates on Large-Scale Proteomics Data Sets with Percolator 3.0

**DOI:** 10.1007/s13361-016-1460-7

**Published:** 2016-08-29

**Authors:** Matthew The, Michael J. MacCoss, William S. Noble, Lukas Käll

**Affiliations:** 1 Science for Life Laboratory, School of Biotechnology, KTH – Royal Institute of Technology, Box 1031, 17121 Solna, Sweden; 2 Department of Genome Sciences, School of Medicine, University of Washington, Seattle, WA 98195 USA; 3 Department of Computer Science and Engineering, University of Washington, Seattle, WA 98195 USA

**Keywords:** Mass spectrometry - LC-MS/MS, Statistical analysis, Data processing and analysis, Protein inference, Large scale studies

## Abstract

Percolator is a widely used software tool that increases yield in shotgun proteomics experiments and assigns reliable statistical confidence measures, such as *q* values and posterior error probabilities, to peptides and peptide-spectrum matches (PSMs) from such experiments. Percolator’s processing speed has been sufficient for typical data sets consisting of hundreds of thousands of PSMs. With our new scalable approach, we can now also analyze millions of PSMs in a matter of minutes on a commodity computer. Furthermore, with the increasing awareness for the need for reliable statistics on the protein level, we compared several easy-to-understand protein inference methods and implemented the best-performing method—grouping proteins by their corresponding sets of theoretical peptides and then considering only the best-scoring peptide for each protein—in the Percolator package. We used Percolator 3.0 to analyze the data from a recent study of the draft human proteome containing 25 million spectra (PM:24870542). The source code and Ubuntu, Windows, MacOS, and Fedora binary packages are available from http://percolator.ms/ under an Apache 2.0 license.

**Graphical Abstract:**
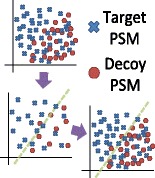
ᅟ

ᅟ

## Introduction

Percolator [1] has played a prominent part in the analysis pipelines of shotgun proteomics experiments for the last decade, as a post-processor of the results from database search engines such as SEQUEST [2], MASCOT [3], X! Tandem [4], and MS-GF+ [5]. Not only does Percolator provide a significant boost in the number of statistically significant peptide-spectrum matches (PSMs) or peptides, it also provides a consistent statistical framework in which to interpret the search results. Because Percolator’s running time is usually much lower than that of the search engine, applying it as a post-processing step should be the default choice when processing shotgun proteomics data. As part of the continuous development and support of the Percolator package, we present two major additions aimed at supporting analysis of large scale proteomics studies.

First, as advances in technology continue to reduce the cost and effort needed to carry out shotgun proteomics experiments, the amount of data per study will keep rising steadily. Although previous versions of Percolator are able to process the data from the vast majority of current studies in a reasonable time frame, the algorithm has some limitations for laboratories without access to a high-performance computing facility. When processing millions of PSMs, the majority of Percolator’s processing time is spent on training support vector machine (SVM) classifiers. In some settings, however, the performance of the SVM as a function of the size of its training set plateaus at a relatively low number of input PSMs [6]. Here, we use Percolator’s semi-supervised learning algorithm to train SVMs on a randomly selected subset of the PSMs and use the resulting score vectors to evaluate the rest of the PSMs. This random downsampling approach yields much shorter analysis times without any loss in statistical power.

Second, we have investigated efficient ways to obtain protein-level accuracy estimates. One of the major obstacles was the question of how to deal with shared peptides and protein grouping. An implementation of Fido [7], which has been part of the Percolator package since 2012, addresses these two issues but is too computationally expensive to apply to large-scale data sets. We therefore compared four straightforward and scalable protein inference methods: using the best-scoring peptide, the two-peptide rule [8,9], the product of peptide-level posterior error probabilities (PEPs), and Fisher’s method for combining independent *p* values.

Although each of these methods is efficient to compute, they each offer specific pros and cons. Savitski et al. [10] showed that on large-scale data sets, taking the best-scoring peptide as the representative of a protein was superior to incorporating information from lower-scoring peptides. However, this approach is unsatisfactory because the method discards all information but the best-scoring PSM for each protein. A simple way to combine evidence at the peptide level is the widely used two-peptide rule. This approach requires evidence for a second peptide to support a protein inference, thereby preventing so-called “one-hit wonders” (*i.e.*, cases where a single, potentially spurious PSM yields a spurious protein detection). An alternative that takes into account even more evidence is to compute the product of peptide-level PEPs. This procedure takes into account all peptides within a protein and provides some protection against one-hit wonders [11]. The method also has the benefit that correctly inferred proteins are not strongly affected by incorrectly inferred peptides because these typically contribute a multiplicative term that is close to 1.0. However, a concomitant drawback to using the product of PEPs is that it is not clear how to scale the resulting product to take into account protein length. Also, it is not obvious a priori that the independence assumption implicit in taking the product applies in this case. The latter concern also applies to Fisher’s method, which is a classic technique for combining independent *p* values [12]. Like the product of PEPs, Fisher’s method takes into account all peptides of a protein, penalizing one-hit wonders on the basis of their many accompanying incorrect peptide inferences [13–15]. This last characteristic can, however, also be a disadvantage, as many incorrect peptide inferences can overrule a minority of correct peptide inferences. Unlike the product of PEPs, Fisher’s method explicitly accounts for the number of *p* values being combined and hence normalizes for protein length.

## Methods

We downloaded three sets of spectra, two from large-scale studies on human samples with several millions of spectra and one smaller-scale study on yeast samples.

The first large-scale set comprises 2212 runs on 17 adult tissues, seven fetal tissues, and six hematopoietic cell types with a total of ∼25 million spectra [16]. The samples were analyzed on an LTQ Orbitrap Velos and Elite (Thermo Scientific) equipped with an Easy-nLC II nanoflow LC systems (Waters). We will refer to this set as the Kim data set.

The second large-scale set was taken from a study aimed at studying variation of protein abundance in humans [17]. It consists of 561 runs on 51 samples from lysates of lymphoblastoid cell lines, resulting in ∼9 million spectra. The peptides were labeled with TMT 6-plex to enable quantification. The analysis took place on an LTQ Orbitrap Velos (Thermo Scientific) equipped with an online 2D nanoACQUITY UPLC system (Waters). We will refer to this set as the Wu data set.

For verification of the accuracy of protein-level false discovery rate (FDR) estimates we additionally downloaded 92, 974 spectra from three injections of yeast cells grown to mid-log phase, collected on an LTQ Orbitrap Velos (Thermo Scientific), as described in Moruz et al. [18]. We will refer to this set as the “hm_yeast” set.

Converting the RAW files to two separate files in the MS1 and MS2 formats [19], respectively, was done with ProteoWizard [20] with vendor peak picking for the MS2 spectra and all other options left at their default values. Next, we assigned high-resolution precursor masses and charges using information from the precursor scans with Hardklör [21] followed by Bullseye [22], both with the default parameters, through the Crux 2.0 package interface [23].

For the Kim data set, the data was searched against the human Swiss-Prot and Swiss-Prot+TrEMBL databases (http://www.uniprot.org/, accessed: November 12, 2015) concatenated with a database of common contaminants (source: http://maxquant.org/contaminants.zip, accessed: April 17, 2015) using the Tide search engine, again through the Crux interface. We used semi-tryptic searches and Tide’s default fragment tolerance. The other search parameters were kept the same as in [16] (10 ppm precursor window, up to two missed cleavages, up to two oxidations of methionine per peptide, variable acetylation of N-termini), except that we did not include variable modifications for the cyclization of N-terminal glutamine.

For the Wu data set, we searched the spectra against the IPI Human database ver. 3.74 (http://www.ebi.ac.uk/IPI, accessed: May 22, 2014) using the Tide search engine through the Crux interface. We used Tide’s default fragment tolerance, and the other search parameters were kept the same as in [17] (10 ppm precursor window, up to two missed cleavages, up to two oxidations of methionine per peptide, variable TMT labeling (229.16293 Da) of lysine and N-terminal amino acids).

The target protein sequences were reversed to construct a decoy protein database, and separate searches were done on the target and decoy protein database for input to Percolator 3.0. We calculated protein-level FDR estimates using the picked target-decoy strategy for all methods [10]. Unless stated otherwise, Percolator was run with the default parameters, which includes target-decoy competition on the PSMs.

### Subset Training

By default, Percolator’s semi-supervised learning algorithm randomly splits the list of PSMs into three subsets (*i.e.*, the cross-validation bins) and trains three separate SVM classifiers, each trained on two of the three subsets and tested on the remaining subset. Each SVM classifier produces a scoring vector, which can then be used to calculate a new score for each PSM based on its feature set. The final scores are thus calculated using the classifier for which the PSM was in the test set.

To implement subset training, we used a downsampling strategy in which we randomly sample a subset of the PSM. Subsequently, we applied Percolator’s normal training algorithm to this subset, resulting in three SVM classifiers in the same fashion as mentioned above. For each PSM, including those not selected for training, we then calculated its score as the average of the scores from each of the three SVMs. This strategy involves some overlap between training and test sets because PSMs selected as part of the training subset will be evaluated by two SVM classifiers, which were trained on this particular PSM, and will only avoid problems of overfitting when the strategy is carried out on large data sets. This strategy was adopted for simplicity of implementation. In a future version of the code, we will implement a scheme in which we downsample each cross-validation bin individually.

Preliminary results showed that including target and decoy PSMs belonging to the same spectrum together during the selection of the random subset gave more stable performance than sampling without taking this distinction into account. Therefore, this strategy was applied in the random sampling process.

### Protein Inference Method Calibration Benchmark

We assessed the accuracy and stability of FDR estimates on the hm_yeast data set by using a *sample* and *entrapment* database, as previously described [14]. The goal of this approach is to provide a ground truth regarding the correctness of peptide and protein inferences made by the algorithm under investigation [24]. The sample database contains the protein sequences of interest, whereas the entrapment database consists of proteins in which the peptide sequences in the sample database are shuffled. We search against the concatenated database of the sample and entrapment database, and subsequently assume that any match to the sample database is a true positive and any match to the entrapment database is a false positive.

The assumption that any match to the sample database is a true positive is not necessarily true because the peptide or protein could have been inferred through an incorrect PSM. The purpose of the entrapment database is to attract the majority of these incorrect target PSMs, thereby ensuring that the majority of PSMs, peptides and proteins matching to the sample database are correct. The larger the entrapment database, the higher the probability that an assumed true positive (*i.e.*, a match to the sample database) is actually true. For example, using an entrapment database nine times the size of the sample database means that we will underestimate the true amount of false positives in the entrapment FDR by ∼11% on the PSM level. However, under the assumption that many of the proteins in the sample database are in fact present, this underestimation is far lower on the protein level because correct proteins in the sample database will conceal incorrect PSMs matching to it.

Here, the yeast Swiss-Prot database (http://www.uniprot.org/, accessed: March 15, 2016) was taken as the sample database, and the entrapment database was nine times the size of the sample database. Furthermore, we artificially added shared peptides between the sample and entrapment database by keeping 4% of the sample peptides unshuffled in the entrapment database. This corresponded to the shared peptide rate in the original Swiss-Prot yeast sample database.

The Tide search engine was used to obtain PSMs, again through the Crux interface. We did a full-digestion search using trypsin (including cleavage suppression by proline) with no miscleavages, specifically chosen to prevent unintended shared peptides between the sample and entrapment databases. The minimum and maximum peptide lengths were, respectively, set to 7 and 50 amino acids. All other parameters were left at their default values. The procedure for the decoy model was identical to the one mentioned above for the Kim and Wu data sets.

### Protein Inference Method Performance Benchmark

The Kim and Wu data sets were used as an indication of the performance on large-scale data by comparing the number of identified protein groups for different protein-level FDR thresholds for each of the protein inference methods. In addition, to assess the performance for differently sized data sets, we looked at the number of inferred protein groups for random subsets of the Kim data set. Unlike in our resource-saving downsampling described above, we this time reduced the size of the evaluated sets, as our interest this time was to test the performance of the inference procedures on smaller sets of peptides.

## Results

### Percolator Works Well on Downsampled Data

We used the Kim data set to evaluate the robustness of Percolator’s SVM classifier to reductions in the size of the training set. The Tide searches of the complete Kim data set against the human Swiss-Prot database resulted in 73 million target and decoy PSMs. Post-processing the full data set with Percolator resulted in 7,928,454 significant PSMs and 298,301 unique target peptides at a *q* value threshold of 0.01. To characterize the performance of the SVM learning procedure when training on subsets of the PSMs, we evaluated performance using training subsets of 100,000, 500,000, 1,000,000 and 5,000,000 PSMs from the Kim data set, using the subset training procedure outlined in the Methods section. For each training subset size, we calculated the mean and standard deviation over 10 randomized runs of the number of PSMs and peptides with *q* value below 0.01.

**Figure 1:**
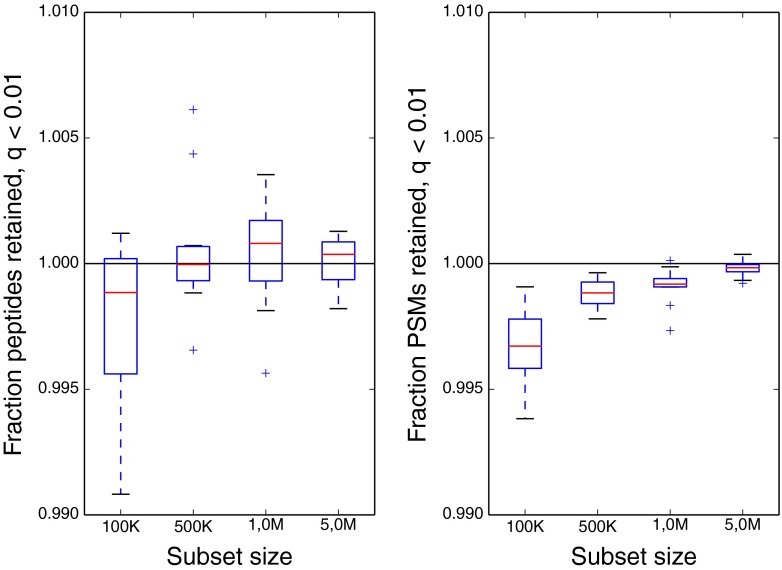
SVM training on downsampled data retains the performance achieved using the full data set. From the full Kim data set of 73 million target+decoy PSMs, we evaluated subset sizes of 100,000, 500,000, 1,000,000, and 5,000,000 PSMs to train the SVMs, repeating this for 10 randomized sets, and scored all 73 million PSMs using the resulting support vectors. The figure plots, as a function of data set size, the ratio of significant peptides (left) and PSMs (right) at a *q* value threshold of 0.01 over the same number when using the full training set of 73 million PSMs. The number of significant PSMs and unique peptides does not drop significantly, even for subsets of 100,000 PSMs

This experiment showed that using subsets as small as 100,000 PSMs (0.14%) for SVM training did not significantly reduce the number of inferred peptides and PSMs (Fig. 1). The standard deviation of inferred PSMs across the randomized runs for a fixed subset size did seem to increase slightly when taking increasingly smaller subsets, but this effect was small. By using a subset of 500,000 PSMs to train the SVM, Percolator’s runtime for producing peptide-level results was reduced from almost a full day to less than 10 min. Furthermore, the memory consumption dropped from almost 100 GB to just 30 GB, allowing analysis of this type of large-scale data to be done on commodity computers.

### Protein-Level FDR Estimates are Poorly Calibrated when Shared Peptides are Retained

We assessed the accuracy of decoy-based FDR estimates derived using four protein inference methods—best-scoring peptide, two-peptide rule, product of PEPs, and Fisher’s method—by analyzing the hm_yeast set. The assessment employed our previously described sample/entrapment strategy [14], which involves comparing the *q* values reported based on the decoy model, the “decoy FDR,” to the fraction of entrapment proteins in the set of inferred target proteins, which we call the “entrapment FDR.”

First, we compared the four protein inference methods while retaining shared peptides. We grouped proteins that had the same set of inferred peptides, and also added proteins whose inferred peptides formed a strict subset of this set to each group [25,26]. We performed the experiment three times, varying the peptide-level FDR threshold (10, 5, and 1%) used during the protein grouping procedure.

**Figure 2:**
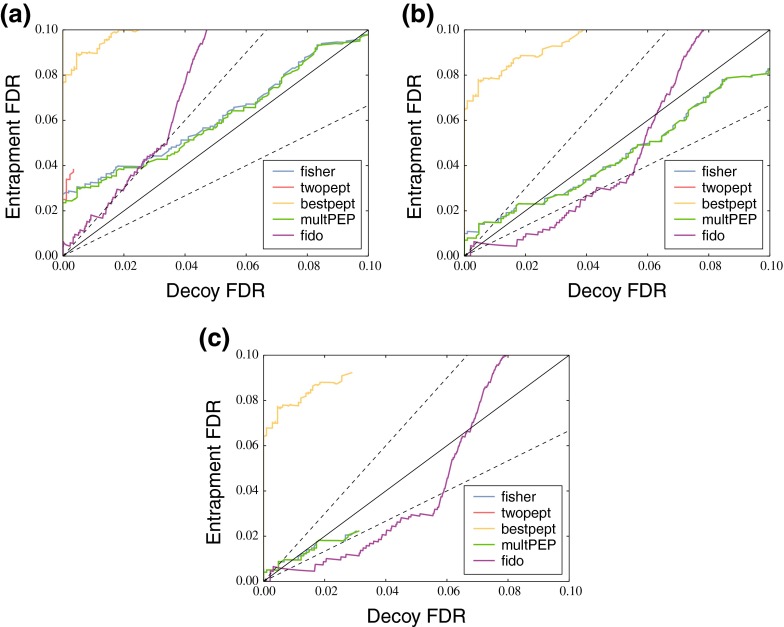
Retaining shared peptides leads to poor calibration of the decoy model for all the tested protein inference methods. The figure plots reported *q* values from the decoy model, the decoy FDR, against the fraction of entrapment proteins in the set of identified target proteins, the observed entrapment FDR using a peptide-level FDR threshold of 10% **(a)**, 5% **(b)**, and 1% **(c)**. Dotted lines correspond to *y* = 1.5*x* and *y* = 0.67*x*. For a peptide-level FDR threshold of 10%, all five methods produce anti-conservative FDR estimates, with Fisher’s method and product of PEPs achieving reasonable accuracy above 3% decoy FDR. For the stricter thresholds of 5% and 1%, the FDR estimates of those two methods are anti-convervative for very low FDRs, but quickly become conservative for higher FDRs. In comparison, the FDR estimates produced by Fido are better calibrated in the very low FDR range, but show rather erratic behavior by suddenly switching from conservative to anti-conservative estimates around 6% entrapment FDR

This experiment showed that the decoy models based on the reversed protein database systematically produce liberal (anti-conservative) FDR estimates (Fig. 2). Fisher’s method and the product of peptide-level PEPs are too liberal for small thresholds but manage to provide better estimates above ∼3% and ∼1% protein-level FDR for the 10 and 5% peptide-level FDR thresholds, respectively. For the two-peptide rule, not enough decoy proteins remain to assess if the FDR estimates will become more accurate at some point, and the best-scoring peptide approach produces dramatically liberal estimates for all thresholds. Taking stricter peptide-level thresholds generally improved the accuracy for Fisher’s method and the product of PEPs. Going down to 5% peptide-level FDR still produced anti-conservative protein-level FDR estimates in the region below 1% protein-level FDR, but going further down to 1% peptide-level FDR actually produced reasonable, though still slightly anti-conservative, estimates in that region.

We also investigated the accuracy of the FDR estimates calculated by Fido, as implemented in the Percolator package, which would still be a viable alternative for smaller data sets. Fido makes use of shared peptides and protein grouping, but contrary to the protein grouping procedure outlined above, only groups proteins with exactly the same inferred peptides. It relies on its Bayesian inference engine to solve the issue of proteins whose peptides form a strict subset of the set of inferred peptides of another protein. Furthermore, Fido estimates FDRs by its Bayesian inference engine rather than using a decoy FDR, although the decoy FDR curve is in fact used to calibrate its parameters. Fido’s FDR estimates proved to be better calibrated in the region below 1% protein-level FDR than the other four methods, but showed divergence from accurate estimates for higher protein-level FDRs, both in conservative and anti-conservative direction.

### Eliminating Shared Peptides Improves Calibration

We hypothesized that these calibration problems arise from the peptides that are shared between different proteins in the database. Accordingly, we next considered an approach that discards shared peptides, retaining only those that are unique to a single protein. To reduce the effect of fictitious shared peptides that correspond to protein fragments or other truncated protein forms, we used the approach to handling shared peptides from Nesvizhskii et al. [27]. Here, proteins are mapped to their theoretical proteolytically digested peptides, rather than their experimentally discovered peptides, and two proteins, *A* and *B*, are merged into a group if protein *A*’s peptides are a superset of protein *B*’s peptides or vice versa. We then retain the peptides that are unique to a protein group rather than to a single protein. A side effect of this change is that in contrast to the procedure used in Fig. 2, we do not have to apply any peptide-level FDR threshold because all protein grouping is done before the data is observed.

We applied this approach to several protein databases and showed empirically that including the protein grouping step increases the number of protein entities (*i.e.*, single proteins or protein groups) with a peptide uniquely identifying it. The experiment involved performing a fully tryptic digestion, with no missed cleavages, of three human protein databases—Swiss-Prot, Swiss-Prot+TrEMBL, and Ensembl—considering peptides with lengths of 6–50 amino acids. TrEMBL and Ensembl contain many proteoforms and therefore benefit significantly from this particular protein grouping approach in terms of number of identifiable protein entities (Table 1). Note that, unfortunately, some protein groups will remain unidentifiable because all their peptides are shared by at least two different protein groups. The in-silico protein digestion and subsequent grouping required only 15 s for the Swiss-Prot database and 4 min for the Swiss-Prot + TrEMBL database.

**Table 1:** Protein Grouping Increases the Number of Inferable Protein Entities

	Swiss-Prot	Swiss-Prot+TrEM	Ensembl
Protein sequences	20,201	69,714	101,933
Peptide sequences	586,424	664,801	672,519
Proteins with protein-specific peptides	19,938	52,834	49,871
Protein groups	20,104	58,605	68,370
Protein groups with protein group-specific peptides	19,978	54,292	58,929

**Figure 3:**
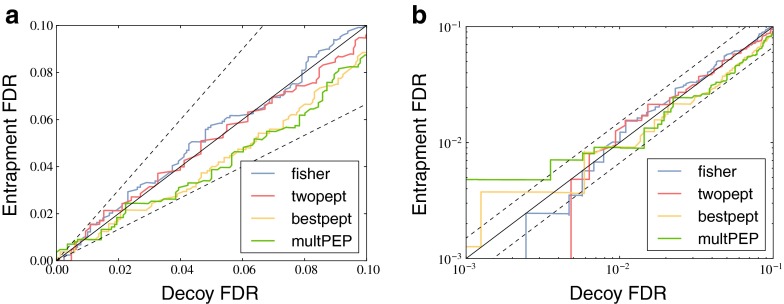
Using only protein-unique peptides gives accurate estimates of the protein-level FDR. **(a)** The figure plots the decoy FDR against observed entrapment FDR. All four methods produce accurate FDR estimates. **(b)** A logarithmic plot of the region [0.001,0.1] with the same axes as in **(a)**

Having eliminated the shared peptides, we returned to our sample/entrapment strategy for estimating the accuracy of protein-level FDR estimates. In this new setting, all four methods now gave accurate protein-level FDR estimates (Fig. 3a). Zooming in on the low FDR region (Fig. 3b) showed that the protein-level FDR estimates break down somewhere in the [0.001,0.01] range, presumably due to the low density of decoy proteins in that region. From these results, it became clear that taking only unique peptides, together with the protein grouping approach from Nesvizhskii et al., would be the most robust choice, regardless of the protein inference method.

### Selection of a Protein Inference Strategy

**Figure 4:**
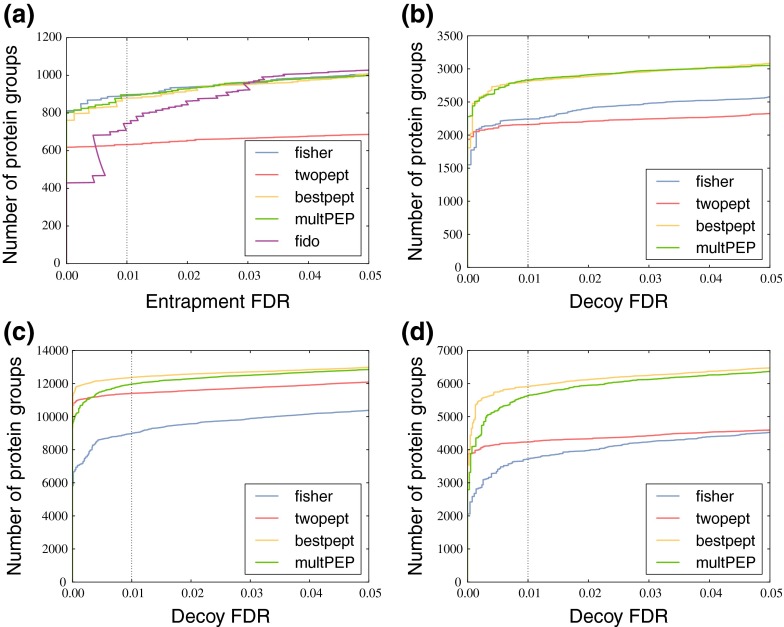
Comparison of protein inference methods. **(a)** The figure plots the number of accepted protein groups against the observed entrapment FDR for the hm_yeast set. Fisher’s method, the product of peptide-level PEPs, and the best-scoring peptide approach all perform about equally, whereas Fido and the two-peptide rule are much less sensitive. We used a peptide-level threshold of 5% for Fido in this plot, but thresholds of 10% and 1% gave very similar results. **(b)** A plot of the number of accepted protein groups against the decoy FDR for the Wu data set. The product of peptide-level PEPs and the best-scoring peptide approach perform best, whereas the two-peptide rule and Fisher’s method inferred far fewer protein groups. **(c) (d)** Same axes as in **(b)** but for the Kim data set, searched using the Swiss-Prot **(c)** and Swiss-Prot+TrEMBL **(d)** databases. The best-scoring peptide approach inferred the most protein groups for both databases

Finally, we compared the number of inferred proteins as a function of the observed entrapment FDR for the different inference methods. For the hm_yeast data set, Fido and the two-peptide rule were clearly performing worse than the others (Fig. 4a). We then repeated the assessment on the much larger Wu and Kim data sets, using the IPI database for the Wu set and two different databases (Swiss-Prot and Swiss-Prot+TrEMBL) for the Kim set. Even for such large-scale data, all four protein inference methods took less than a minute of processing time because of their simplicity. We looked at the number of inferred protein groups at 1% reported protein-level FDR (Fig. 4b–d). For the Wu data set, the multiplication of PEPs and the best-scoring peptide approach performed best. Fisher’s method inferred far fewer protein groups than the other three methods, possibly due to incorrect peptides dragging down the *p* value of correct protein groups. For the Kim data set, the best-scoring peptide approach inferred the most protein groups for both databases with a 3–5% margin over the second-best method. Although the many proteoforms in the TrEMBL database caused a large drop in the number of protein groups for all methods, the relative ranking of methods was consistent in both sets of results.

**Figure 5:**
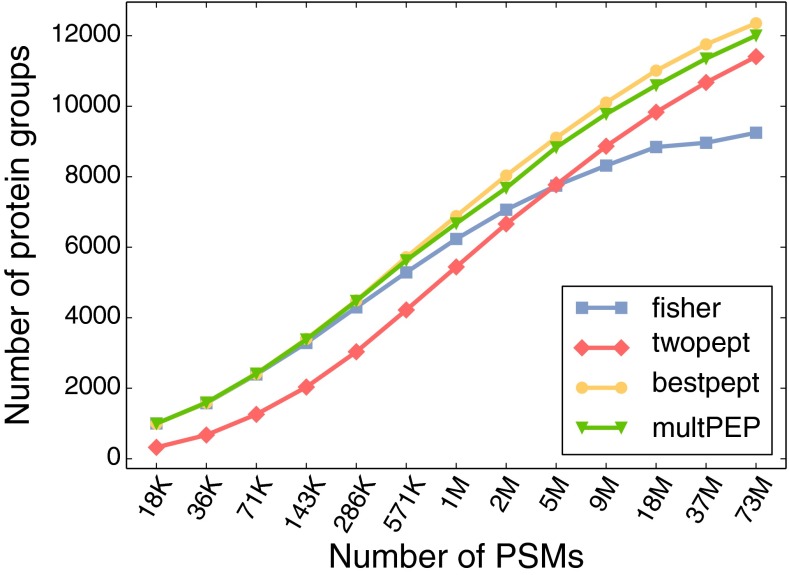
Sample size dependence of protein inference methods. We plotted the average number of accepted protein groups at 1% protein-level FDR over triplicate random subsets of using different subset sizes of the Kim set matched to the Swiss-Prot database. The number of PSMs was reduced from the original 73 million PSMs with factors of two until 18 K PSMs. Fisher’s method, the product of peptide-level PEPs and the best-scoring peptide approach all perform about equally until 200 K PSMs. Above this number of PSMs, the best-scoring peptide approach outperforms all the other methods

Finally, we looked at the number of inferred protein groups for random subsets of the Kim data set (Fig. 5). For small data sets, all methods except the two-peptide rule perform about equally. However, as the data sets get larger, the best-scoring peptide approach starts to show its advantage. Overall, we concluded, in agreement with Savitski et al. [10], that the best-scoring peptide approach yielded the best overall performance. We therefore implemented this protein inference method in the latest Percolator package.

## Discussion

We demonstrated that Percolator 3.0 can calculate accurate protein-level FDRs on a human proteome-scale study, in this case 73 million PSMs, in a matter of minutes on a commodity computer.

The downsampling approach for SVM training on a subset of the PSMs shows great stability even when only sampling a tiny fraction, as small as 100,000 PSMs (*i.e.*, 0.14% of the original set of 73 million PSMs). The Kim data set might not be as representative for other types of studies that have greater heterogeneity, but it seems likely that the downsamping strategy will work well as long as the selected subset of PSMs contains a sufficient number of positive training examples.

The most successful protein inference method turned out to be the one where proteins were grouped by their theoretical peptide sets and only the best-scoring peptide was considered. In this approach, the score assigned to a protein essentially ignores the majority of the PSMs, something that may feel quite unsatisfactory. On the other hand, including evidence from other, lower-scoring peptide inferences is difficult because it often involves lumping incorrect peptide inferences into correct protein inferences. This problem can clearly be seen in the poor performance of Fisher’s method for combining *p* values on the Wu and Kim data sets. Setting peptide-level thresholds can actually bring Fisher’s method up to par with the best-scoring peptide approach (data not shown). However, this modification does not produce significantly more protein inferences, while introducing an extra parameter that needs to be set correctly.

With regards to the discarded shared peptides, large-scale studies give us the luxury of deep coverage, therefore inferring many peptides that are unique to a protein. This mitigates the problem of ignoring shared peptides and makes the task of protein inference much simpler and intuitive.

The protein grouping method employed here still suffers from the problem that an inference of a protein group leaves open the question of which proteins in the group are actually correct. Here, we interpreted it using the null hypothesis that all proteins in the group are incorrect (*i.e.*, an inferred protein group means that we expect at least one of the proteins to be correct) but we are agnostic about which one. Compared with the conventional protein grouping approach, which is based on inferred peptides instead of the full set of theoretical peptides, the groups produced by our method are much smaller. For the Swiss-Prot database, virtually all protein groups contained only one protein and for the Swiss-Prot + TrEMBL database they typically contained just one or two proteins. Consequently, ambiguity about which specific proteins are present is much rarer. Furthermore, if genes, rather than proteoforms, are the entities of interest, using databases with few proteoforms such as Swiss-Prot should do the job.

We have not extensively tested our new protein inference functionality on smaller data sets. However, based on the results for the hm_yeast set and the small random subsets of the Kim set, we believe that the estimated FDRs will remain accurate and that it is unlikely that any of the other evaluated protein inference methods will identify many more proteins.

The construction of the entrapment database in this study should be considered as a rather crude approximation of true experimental settings and could be improved upon in future work. Although our approach does conserve homologs by shuffling peptide rather than protein sequences, the method only allows for simulation of fully-tryptic peptides without missed cleavages. Furthermore, the mechanism that creates shared peptides between sample and entrapment database does not attempt to model homology. In our approach, the shared peptides are randomly distributed over all proteins, whereas in practice we can expect some portion of proteins to share multiple peptides and many proteins to have no shared peptides at all. The rate of shared peptides—4%, modeled after the Swiss-Prot yeast database—is also a good approximation of the shared peptide rate of the Swiss-Prot human database. However, the shared peptide rate in the Swiss-Prot+TrEMBL database is much higher, with over 60% of the peptides being shared by at least two proteins. Modeling this level of redundancy could result in significantly different outcomes.

For our largest evaluated data set, the 73 million PSMs of the Kim data set, the combined runtime for Percolator 3.0 (*i.e.*, from PSMs to protein-level FDRs) was 10 and 15 min for the Swiss-Prot and Swiss-Prot+TrEMBL database, respectively. We realize that other parts of the shotgun proteomics analysis pipeline might still have significantly higher computing requirements than Percolator, but fortunately these can often readily be parallelized as the runs can be analyzed independently. However, as we have pointed out elsewhere, obtaining significance measures per run or data set and combining them afterwards is not at all straightforward and should be handled with great caution [28]. This new version of Percolator allows the user to easily obtain statistical significance measures on aggregated data from a great number of runs without running this risk.
